# Stimuli-Responsive Systems in Optical Humidity-Detection Devices

**DOI:** 10.3390/ma12020327

**Published:** 2019-01-21

**Authors:** Sergio Calixto, Valeria Piazza, Virginia Francisca Marañon-Ruiz

**Affiliations:** 1 Centro de Investigaciones en Óptica, Loma del Bosque 115, Leon C.P. 37150, Gto., Mexico; vpiazza@cio.mx; 2 Laboratorio de Ciencias Químicas/Área de Química Orgánica, Departamento de Ciencias de la Tierra y de la Vida, Universidad de Guadalajara, Centro Universitario de los Lagos, Enrique Diaz de León 1144, Col. Paseo de la Montaña, Lagos de Moreno C.P. 47460, Jalisco, Mexico; vmaranon@culagos.udg.mx

**Keywords:** stimuli-responsive materials, gelatin, interpenetrated polymers, relative humidity, diffraction gratings, permeability, climatic chamber

## Abstract

The use of electronic devices to measure Relative Humidity (RH) is widespread. However, under certain circumstances, for example when explosive gases are present, a spark-free method should be used. Here we suggest the use of stimuli-responsive materials, like gelatin and interpenetrated polymers, to detect RH with an optical method. These materials are hydrophilic. When water vapor is absorbed by the films the molecules attach to the films molecular network. The result is that the film thickness increases and their refractive index changes. To detect the change of these two parameters an optical method based on diffraction gratings is employed. Surface diffraction gratings are recorded on the films. Then gratings are placed in an optical configuration that is immersed in a climatic chamber. A light beam is sent to the grating where it is diffracted. Several light orders appear. Due to the absorption of water molecules the films swell and grating surface modulation changes. This implies that the diffracted orders intensity changes. A calibrating plot relating intensity as a function of RH is obtained.

## 1. Introduction

Stimuli-responsive polymers present changes when subjected to external parameters like pH, temperature, solvent composition, radiation, mechanical stress, light, electrical and magnetic fields, and chemical triggers like glucose to mention but a few. These stimuli can trigger discontinuous changes (phase transitions) or gradual changes that occur over a finite range of stimulus levels. The changes could be reversible but hysteresis may occur. In recent years, new, smart polymers with dynamic properties were designed, by exploiting either reversible covalent bonds or supramolecular interactions to induce topological changes in chain structure [1,2].

In general stimuli-responsive materials can be used in sensors, drug delivery carriers, artificial muscles and more [3]. Among the key properties of stimuli-responsive materials for most applications is their degree of swelling, swelling kinetics and permeability. The swelling degree (i.e., the ratio of wet mass to dry mass) is important because it affects the kinetics, permeability and modulus. Among other factors, the swelling of stimuli-responsive materials is a function of crosslinking junction concentrations. Swelling kinetics and permeability are related. Increasing the swelling degree dilutes the network and this leads to an increased permeability to solutes. Conversely, a decrease of the swelling degree gives a decrease in permeability. Analysis of the swelling or shrinking kinetics of materials is primary for the design of the stimuli-responsive applications. Transparency can be a key for some applications, such as light sensors. Other key properties could be biocompatibility and biodegradability when stimuli-responsive materials have medical and pharmaceuticals applications.

The word humidity [4] is used when we are dealing with water vapor which is a gas. Water vapor is present in the earth’s environment. Humidity measurements are important in industry, laboratory and common life activities. Thus humidity instrumentation that met rigid requirements has been developed. There are many types of humidity sensors called hygrometers. For every measurement the most useful sensor should be selected considering its high sensitivity, short response time, linear response, long term stability, small hysteresis, good durability, resistance against contaminants, cost, maintenance, calibration requirements, ease of installation and service to mention but a few. Besides this there are several techniques to measure humidity. Because there is no humidity sensor available that can cover the full dynamic range of water vapor levels different measurement methods and sensors have been developed each having certain advantages and limitations and suitable for some, but not all, applications. The most fundamental measurement technique of humidity is the chilled mirror hygrometer [5]. Other techniques comprise optical fibers [5], Micro Electromechanical Systems (MEMS) [6], hygrometers based on paper [7] and poly(vinylalcohol) (PVA), nanocomposites [8] containing graphene, among many others. Hygrometers have sensors that interact with water vapor by absorption, adsorption, and desorption of water molecules. Absorption could be by diffusion and capillary action.

In this paper we present a continuation of the work that we have developed and presented before [9,10]. In Section 2 we describe the materials used to develop the sensor in the form of films and the method to fabricate them. Section 3 shows the Relative Humidity (RH) characterization of films behavior. Section 4 describes the gratings theoretical background. Section 5 shows the gratings optical fabrication method and the study of gratings profile. Section 6 describes the climatic chamber and calibration method to quantify RH by means of the diffracted orders intensity. Section 7 deals with hysteresis shown by the films. Finally in Section 8 conclusions are drawn. 

## 2. Materials

### 2.1. Gelatin, PVA and Poly(Acrylic Acid) (PAA)

Gelatin is a derived protein that does not occur free in nature. It is primarily used in the food and pharmaceutical industries as a gelling agent. The colorless gels that gelatin forms, in the presence of an appropriate solvent, are thermoreversible when cooled below about 35 °C. 

For gelatin production, collagen from porkskins, cattle bones or hides is hydrolyzed and extracted either with an acid or alkaline pre-treatment, generating the Type A and Type B products, respectively. Both types of gelatin consist of a heterogeneous mixture of single or multi-stranded polypeptides containing less than 1000 amino acids. This is due to the partial breakage of collagen and the renaturation of the collagen-like helical structure among some of these fragments. 

The aminoacidic chains of gelatin are typically enriched in glycine (approximately 30% of the aminoacidic content), proline and 4-hydroxyproline residues. These aminoacidic chains display several groups that can form hydrogen bonds with water, predominantly the hydroxyproline hydroxyl groups and the peptide carbonyl groups.

The properties of gelatin depend heavily on its water content: If dehydrated below a 2% moisture level, it becomes insoluble in water because of crosslinking between the gelatin macromolecules. Dehydrated gelatin is thus a crosslinked polymer, extremely brittle in the solid state. Gelatin undergoes transformation to a rubbery state at around 25% moisture content and at room temperature. 

These and other physical properties of gelatin might be correlated to the different states in which water is present inside the gel. Similarly to what is found in other protein mixtures or polymers, water inside gelatin can be free, weakly bound (also called intermediate water) or tightly bound [11]. The degree of water molecules association with the chains of the polymer, in this case with collagen helices, determines also the physical behavior of water itself: The water molecules that establish the strongest bond with the polymer lay in close proximity to the aminoacidic chains and are unable to freeze, while intermediate water molecules interact less tightly with the chains and undergoes cold crystallization, i.e., freezes at temperatures below 0 °C. Amidst the aminoacidic chains, free water is able to move and freezes at 0 °C; it is the first to leave gelatin when dehydration occurs. When the water content drops below 15–20%, virtually no free water is present inside the polymer [12].

Chemical structure of gelatin films can be modified by means of ultraviolet light, heat and some chemicals. For example if a blend of gelatin and dichromates is illuminated with UV light, crosslinking will occur in the gelatin network. Regarding chemicals the use of formaldehyde can harden the gelatin. Commercial photographic fixing baths contain some chemicals like sodium or ammonium thiosulfate, acetic acid, sodium sulfite and potassium alum. This last chemical will form more crosslinks in the gelatin network preventing excessive gelatin swelling or softening.

Poly(vinyl alcohol) PVA is an atactic material that exhibits great crystallinity; in structural terms it has 1,3-diol bonds (–CH_2_–(OH)–CHCH_2_–(OH)CH–)_n_. It has been widely used for its emulsifying and adhesive properties, but its main feature related to this work is its ability to absorb water, which is why it is considered a hydrogel. The water molecules act as plasticizers, which reduces the tensile strength of the polymer and increases its elongation allowing its resistance to tearing. The permeation of oxygen through the hydrogel is substantially facilitated when there is equilibrium of water content with PVA. This polymer absorbs and retains large amounts of water molecules [13]. 

Poly(acrylic acid) (PAA) was used as a crosslinking reagent because it has a functional carboxyl group (–CH_2_–(CO_2_H)CH–)_n_ in each monomer unit to react with PVA. Some characteristics shared by both polymers include a high solubility in water and a high miscibility of PAA with PVA. Both polymers are widely commercialized and readily available. They can form a strong crosslinking by an ester bond between the hydroxyl group of PVA and the carboxyl group of PAA. The crosslinking is caused by strong hydrogen bonds between carboxylic acid of PVA and the hydroxyl groups of PAA. Finally, both polymers are good hydrogels agents [14].

### 2.2. Interpenetrated Polymers

Some materials are fragile and break easily. To strengthen them they are polymerized and crosslinked with other polymers [15,16]. Combination of the two polymers can be prepared in the form of blends, copolymers and interpenetrating polymer networks (IPNs) or interpenetrated hydrogels (IPHs). The IPNs are a combination of at least two polymer chains each in the network form. They form polymer gels held together by permanent entanglements. One polymer is synthesized or crosslinked independently of the presence of the other. Polymers are concatenated and cannot be pulled apart, but they are not bonded to each other by any chemical bond. 

To fabricate the hydrogels the following chemicals, purchased from Sigma Aldrich (St. Louis, MO, USA), were used. The acrylic acid (AA) had a M_w_ 72 g/mol with a purity of 99%. The poly(vinyl alcohol) (PVA) had an M_w_ between 89,000–98,000 g/mol, 99% hydrolyzed. N-N’ methylene bis (acrylamide) with an M_w_ 154 g/mole, purity 99%, was used as a crosslinker, and potassium persulfate was used as a thermal initiator. PVA/PAA IPN films were fabricated following the method described in references [17,18].

### 2.3. Thin Films Fabrication Method

**Figure 1:**
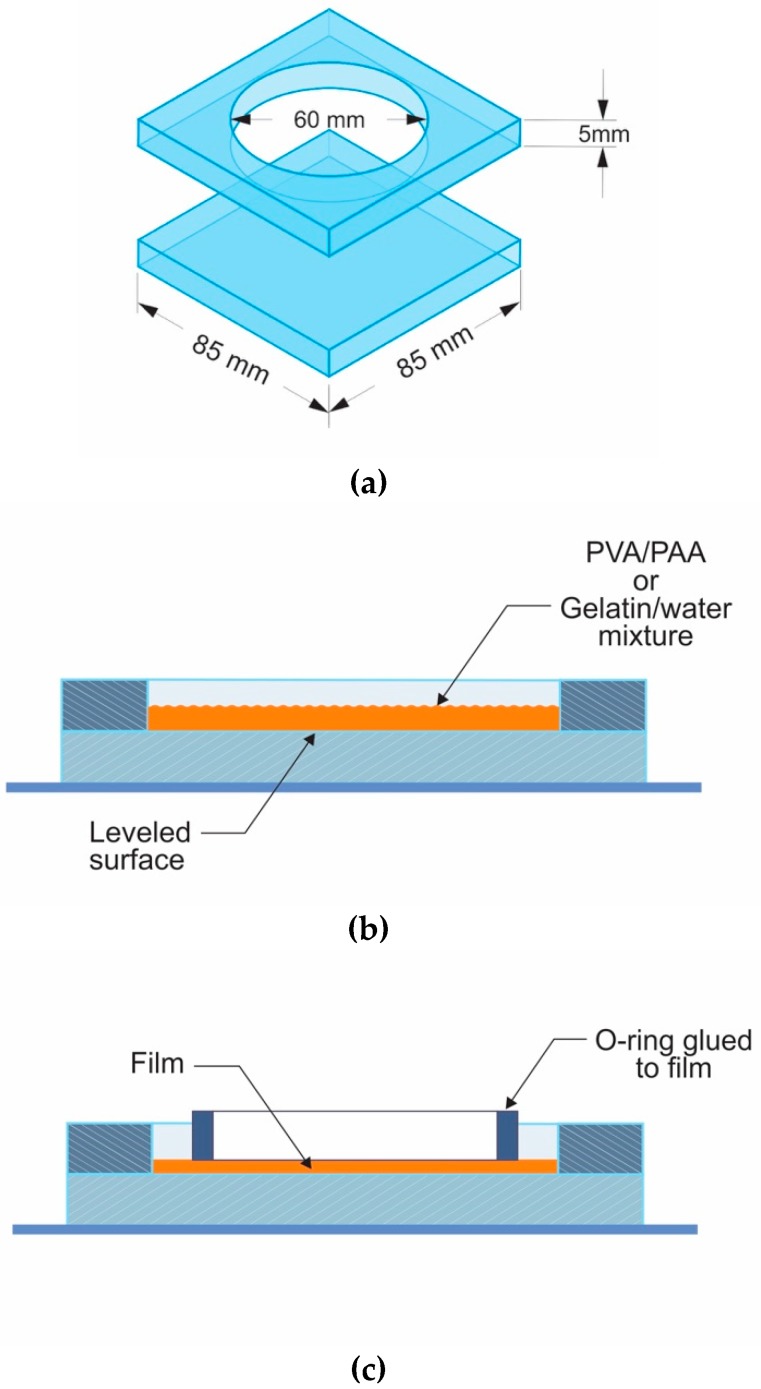
Schematic shows the process to make thin films: (**a**) two glass pieces; (**b**) pouring the mixture and (**c**) gluing the metallic ring.

The materials to be used as sensors should be available in transparent and thin film form. To make the films the surface of two glass plates of about 8.5 cm × 8.5 cm were polished and a hole of about 6 cm diameter was made in one plate, Figure 1a). The plates were placed in contact and the set was leveled. In the hole a mixture of gelatin and water (200 mg of gelatin in 10 mL of water) or PVA/PAA was poured, Figure 1b). After about 24 h a thin film was present. The amount of poured mixture is related to the film thickness. After the film was made a metallic ring was glued to the film, Figure 1c). Then the ensemble was detached from the glass.

## 3. Thin Films Characterization Methods Related with Water Absorption and Permeability

All the experiments described in this article were performed at room temperature. Films used as the active sensor for RH measurements were characterized for their water absorption and permeability characteristics.

### 3.1. Behavior of Films Weight as a Function of Water Molecules Absorption

Water absorption or desorption affects films weight. As we have seen in Section 2 when water molecules are absorbed by the films they interact with the chains of molecules that compose the films. The result is that the weight of the film increases. The opposite happens when the molecules are desorbed. Several materials were tested with the help of a microbalance.

Gelatin and PVA/PAA films were made with a diameter of 6 cm and different thicknesses. Then they were kept in a box that had silica gel for about two hours. After this they were placed in a microbalance and the behavior of their weight as a function of time was found. In the course of time water molecules in the atmosphere were trapped by the films and their weight increased.

**Figure 2:**
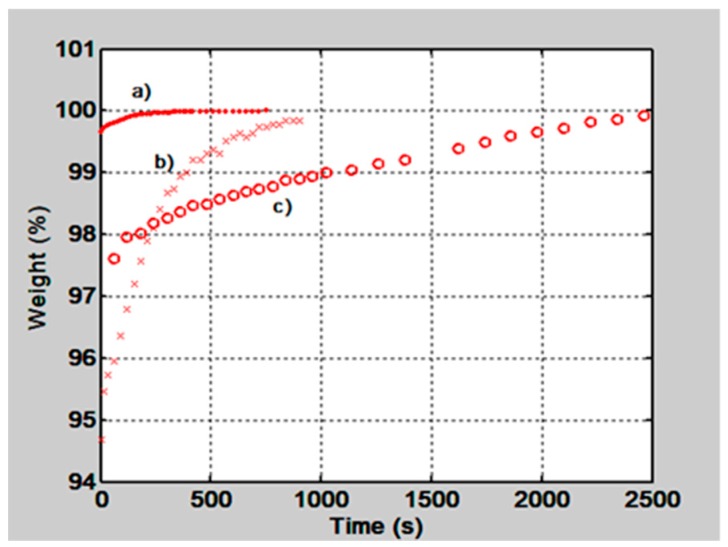
Normalized films weight as a function of time when they were placed in a microbalance and let them absorb environmental water molecules. Plot a) is for a gelatin film with a thickness of 15 µm, plot b) for a 50 µm film and plot c) for a poly(vinyl alcohol) (PVA)/poly(acrylic acid) (PAA), 50 µm film.

In Figure 2 the behavior of the film’s normalized weight as a function of time is shown, for two gelatin films with thickness of 50 µm and 15 µm and for a PVA/PAA film, with a thickness of 50 µm. The gelatin film with 15 µm thickness absorbs quickly the water molecules and reaches the equilibrium state in about 300 s. The 50 µm gelatin film tends to stabilize in about 700 s and the PVA/PAA in about 2500 s.

**Figure 3:**
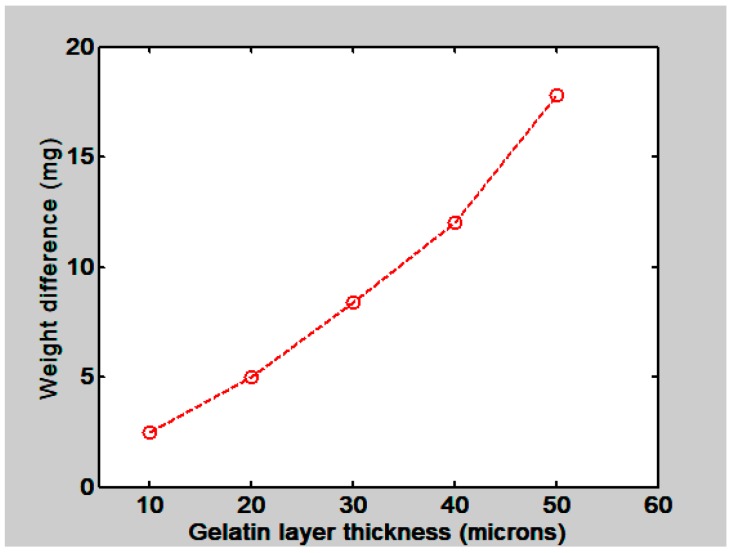
Plot shows the weight difference (mg) between the values when the films were dry and after they had absorbed water molecules from the atmosphere as a function of the film’s thickness (µm).

To find the behavior of gelatin films water absorption as a function of the film’s thickness some films were made. They had the following thicknesses: 10, 20, 30, 40 and 50 µm. After fabrication they were kept in a box with silica gel for about two hours and then they were placed in the microbalance one at a time. During this weighing process they absorbed water molecules from the atmosphere. Plots like the ones shown in Figure 2a),b) were obtained. Then to know the amount of water that was absorbed by the films the weight values at the beginning and at the end of the process were taken from each plot. The one at the end was taken when the plot reached the equilibrium state. The difference between these two measurements gave us the amount of absorbed water, in mg. These values are plotted as a function of the film’s thickness in Figure 3. We can notice that as the film thickness increases the film absorbs more water.

### 3.2. Hardened and Unhardened Gelatin Films

**Figure 4:**
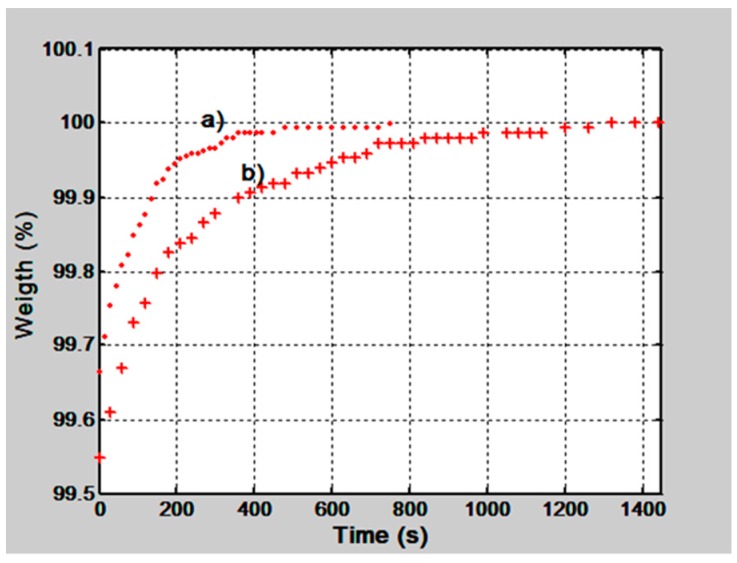
Behavior of the normalized weight of a gelatin thin film, 15 µm thickness, when it was in its unhardened state a) and hardened one b).

It has been mentioned in Section 2.1 that gelatin thin films are hardened by means of UV and visible light or by means of chemical methods. Hardened films will be less hydrophilic because molecular chains will present more crosslinks. We have tested this statement in the following way. A 15 µm thickness gelatin film was made and its water absorption behavior was tested by weighting the film through time. This behavior can be seen in Figure 4, plot a). Then the film was hardened with a photographic film hardener for 3 min [19]. A wash bath for 4 min followed. After drying, the film was placed in a container with silica gel for 2 h and then the weight test was done. The result is shown in Figure 4, plot b). We can notice that plot a), for unhardened film, reaches stability after about 450 s, however, plot b), for the hardened layer, reaches stability after 1200 s. Thus, the film affinity to water molecules has been reduced by hardening it or its sensitivity to RH was diminished.

### 3.3. Films Water Vapor Permeability

**Figure 5:**
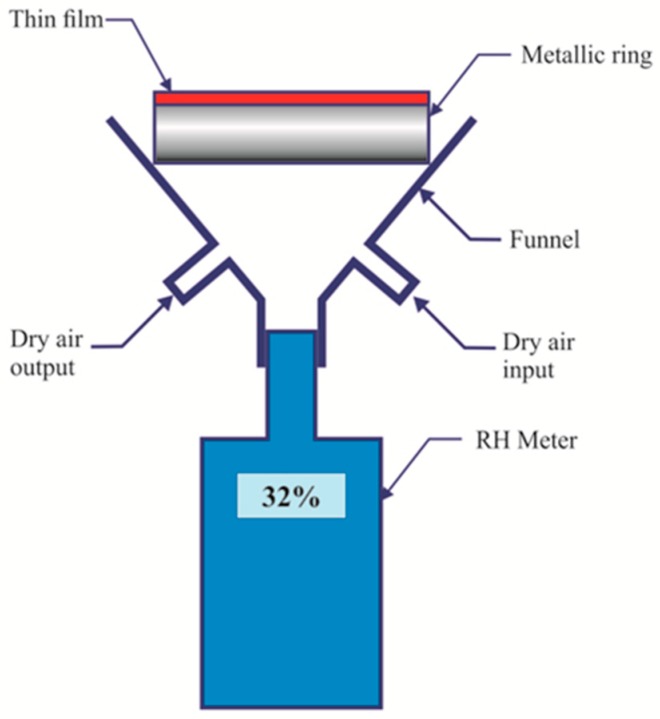
Schematic of the device used to find the film’s water vapor permeability.

In material science films permeability is tested through strict rules or methods. Some of them are mentioned in the ASTM E96 document (Standard tests methods for water vapor transmission of materials) [20]. This test method covers the determination of water vapor transmission (WVT) of materials through which the passage of water vapors may be of importance. Other methods have been exposed [21,22]. An alternative method to measure the films water vapor transmission was developed in our laboratory. A schematic of the device can be seen in Figure 5. An ordinary RH electronic gauge was coupled to a funnel. On top of the funnel a metallic ring was glued to the funnel with silicon. The metallic ring had a thin film glued to it. The funnel had an entrance hole and an exit one. Through the entrance dry air was pumped until a certain RH value (~11%) was reached. During this time the exit hole was opened, i.e., dry air passed freely taking out the water molecules. When the desired low RH was reached, pumping stopped and then the exit hole was closed.

**Figure 6:**
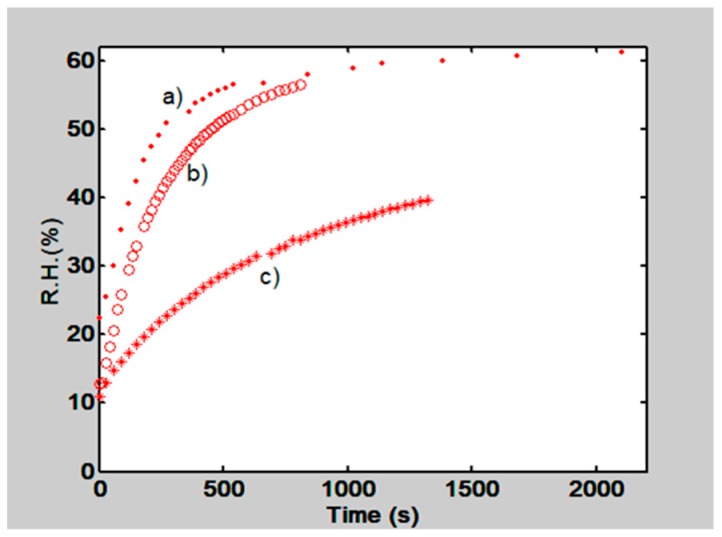
Behavior of Relative Humidity (RH) as a function of time when two gelatin films, a) and b), and a PAA/PVA film, c), were tested. Gelatin films presented thickness of 15 µm a) and 50 µm b). c) shows the behavior for a PVA/PAA, 50 µm film.

Through time the water vapor, present in the atmosphere, began to permeate through the film and the RH measurements were made. Plots in Figure 6 show the behavior of the RH as a function of time for two gelatin films. a) for a 15 µm thick and b) for 50 µm thick film. Moreover, a PVA/PAA film, with a thickness of 50 µm was tested, plot c). From the plots we can see that gelatin thin films let the water molecules pass more easily, while PVA/PAA films hinder the permeation of water molecules. 

### 3.4. UV-Vis and FTIR-ATR Characterization

**Figure 7:**
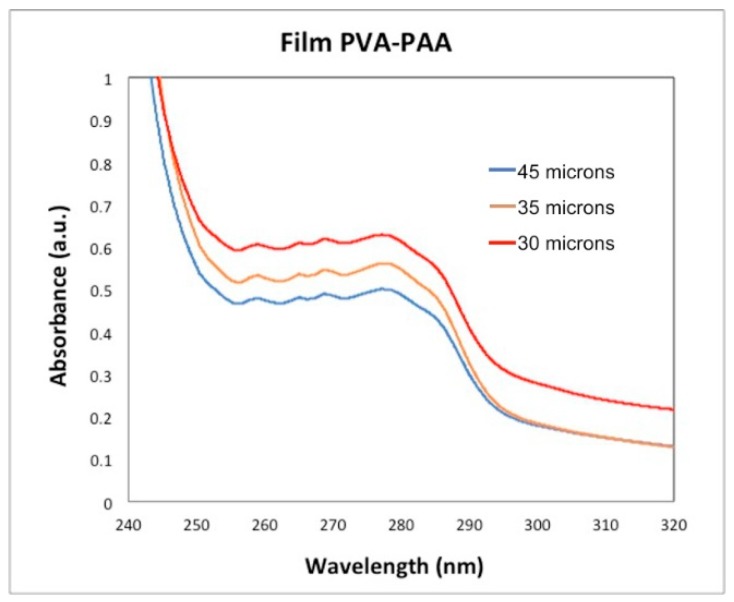
Absorbance as a function of wavelength for three PVA/PAA films with different thickness.

**Figure 8:**
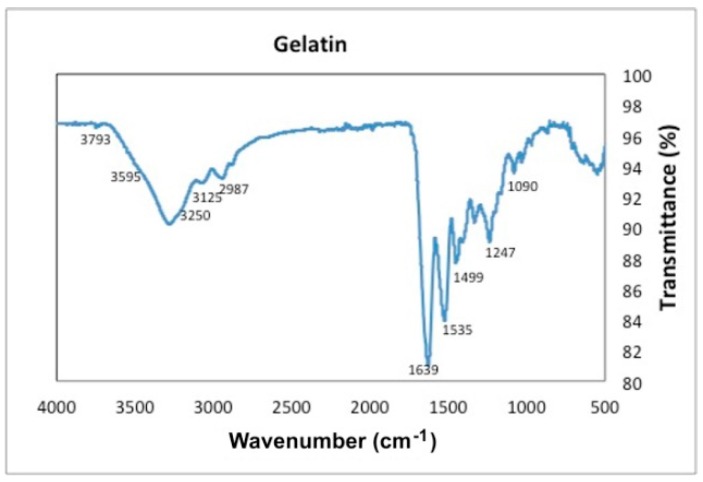
Transmittance as a function of wavenumber for gelatin.

**Figure 9:**
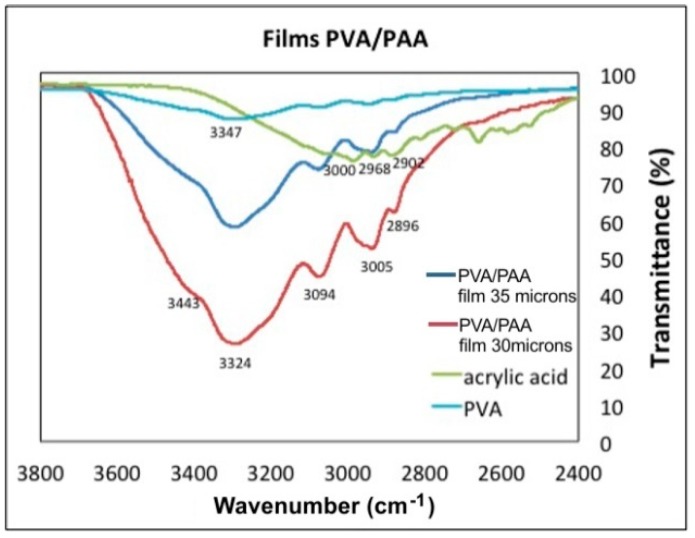
Transmittance as a function of Wavenumber for PVA, PAA, and two PVA/PAA films with different thickness.

**Figure 10:**
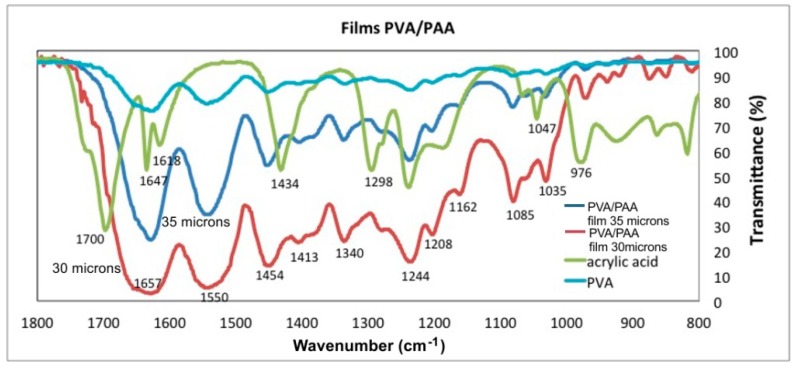
Transmittance as a function of wavenumber for PVA, PAA, and two PVA/PAA films.

The UV-Vis and FTIR-ATR studies are applicable to the chemical characterization of polymers. It is a method that determines the crosslinking between polymers due to the formation of new bonds. The modes of vibration of the bonds between the atoms are differentiated according to the plots displacement in the wavenumber axis. The chemical structure of Gelatin, PAA, PVA and PVA/PAA films was determined with UV-Vis and FTIR spectra presented in Figure 7, Figure 8, Figure 9 and Figure 10. The measurements were performed with a Lambda 365 and a Frontier spectrometer (Perkin Elmer, Waltham, MA, USA).

The UV-Vis spectra of the PVA/PAA films, with 30, 40 and 45 µm thickness, are shown in Figure 7. It is seen that when the thickness increases also the absorbance does. There is a maximum at 278 nm corresponding to the electronic transitions n → σ*, while the electronic transitions σ → σ* are presented between 250–270 nm. The absorbance is a function of the films water molecules thickness inside the PVA/PAA films. 

Table 1 presents the vibrational modes of bands given by gelatin, PVA, AA, and PVA/PAA films.

The FTIR-ATR spectrum of gelatin, Figure 8, shows the characteristic peak at 3250 cm^−1^ given by the hydroxyl group (–OH). The bending at 3595 cm^−1^ given by the amine group OH is also observed. At 3125 cm^−1^ the characteristic vibrations of (–CH) groups are seen. The 2987 cm^−1^ peak corresponds to the symmetric vibrations of the protein (C–H). The stretching vibration from aromatic atoms can be seen between 2392 cm^−1^–1950 cm^−1^. The peaks at the wavenumbers 3250 cm^−1^, 1639 cm^−1^ and 1535 cm^−1^ are attributed to the free water molecules or amides III, II and I respectively. The bend at 1650 cm^−1^ corresponds to the CO stretching. The bend at 1499 cm^−1^ can be attributed to weak NH bending and C–N stretching of amide II and the bend at 1247 cm^−1^ to weak KC–N stretching and NH bending of amide II. Finally, the bends at 1090 cm^−1^ and 1025 cm^−1^ represent the weak bending and C–N stretching of amide III.

Figure 9 and Figure 10 show the FTIR-ATR spectra of PVA, PAA standards and the PVA/PAA films with a thickness of 30 µm and 35 µm. Figure 9 shows the wavenumber range between 3800 cm^−1^ and 2400 cm^−1^ and Figure 10 from 1800 cm^−1^ to 800 cm^−1^. In Figure 9 the PVA plot shows a peak at 3347 cm^−1^ corresponding to the vibrations of hydroxyl bonds (–OH), which is not intense. For the AA plot at 3000 cm^−1^, a wide and displaced signal appears and corresponds to the hydroxyl vibration modes (–OH) of the acid group. When the PVA/PAA crosslinked films are made, a very wide band centered at 3324 cm^−1^ appears and indicates the films ability to absorb water molecules into the polymer crosslinked network. As the thicknes of the PVA/PAA films increases, the range of water vibration modes (H–O–H) becomes wider and more intense (3443 cm^−1^) due to the absorption of water molecules into the crosslinked network. In the 35 µm film the peaks at the wavenumbers 3000 cm^−1^, 2968 cm^−1^, and 2902 cm^−1^ are shown. These vibrations correspond to the asymmetric and symetric stretching vibrations of –CH. However, when the films are formed with the crosslinked PVA/PAA polymer, the vibrations are lost because the new C–C bonds of the interpenetrated hydrogel are formed at 2896 cm^−1^. The plot for the 30 µm thick film shows the characteristic bond signals (=CH) of the vinyl group that appear at 3005 cm^−1^. Finally it can be seen in the PVA/PAA plots that the thinner the film thickness the transmittance at around 3324 cm^−1^ decreases because there is a greater absorption of water molecules [23].

Regarding the wavenumber range between 1800 cm^−1^ and 800 cm^−1^, Figure 10, the PAA plot presents peaks at 1700 cm^−1^, 1647 cm^−1^ and 1618 cm^−1^ that correspond to the vibration modes of carbonyls (C=O). The vibrations modes at 1298 cm^−1^ and 1244 cm^−1^ corresponds to the C–O bond. Concerning the PVA/PAA films the hydrogel intensifies the signal from 1657–1618 cm^−1^, relative to the PAA plot, and a new signal appears at 1550 cm^−1^, both signals correspond to the vibration modes of the new carbonyls (C=O) derived from the PAA forming the networks of cross-linking of the polymer as ester vibration. The peaks at 1657 and 1550 cm^−1^ are broad and intense because the water molecules are linked by blending the cross-linked PAA chain [24]. 

## 4. Optical Principle of RH Sensing Method Using Stimuli-Responsive Polymer Systems. The Diffraction Grating

**Figure 11:**
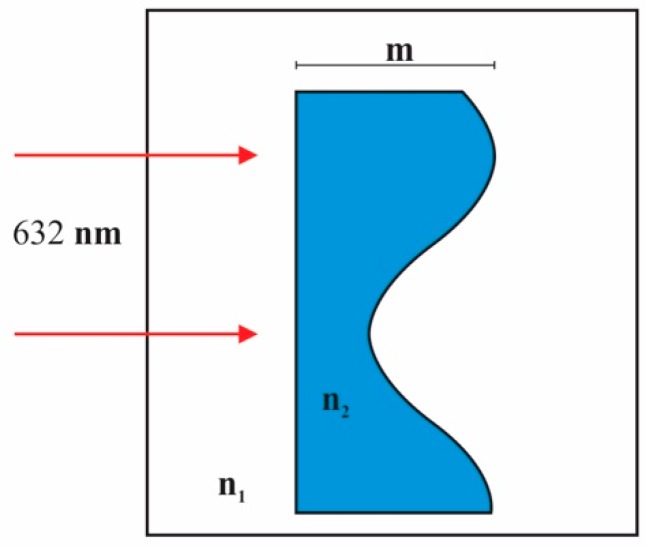
Sinusoidal grating profile.

Theoretical grating behavior can be described by applying the thin phase screen approximation [25]. A grating with a sinusoidal profile is considered, Figure 11.

The grating has a refractive index n_2_ and it is immersed in a medium with refractive index n_1_. Grating shows a deep modulation m. Illuminating light has a wavelength of 632 nm. After light passes the grating some spots appear due to the diffraction (diffracted orders).The first order intensity is described by the following formula:

$$I_{1}\propto {\left[J_{1}\left(\frac{2\pi \;m}{2\;\lambda 0}\left(n_{1}-n_{2}\right)\right)\right]}^{2}$$

In the present study the grating is immersed in the air, so n_1_ = 1. PVA/PAA interpenetrating polymer and gelatin have refractive indices of 1.486 and 1.5, respectively. They were measured with an Abbe refractometer (Abbe refractometer, Bausch and Lomb, Rochester, NY, USA). 

**Figure 12:**
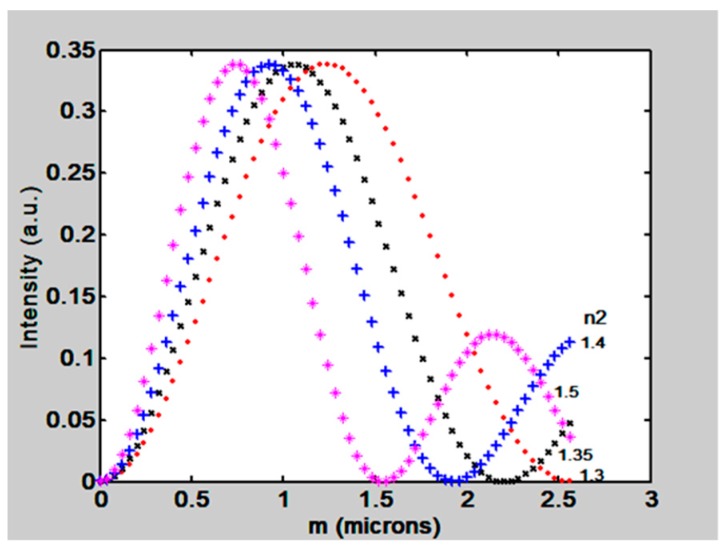
Theoretical behavior of the first order intensity as a function of grating relief modulation m. The refractive index n_2_ presented several values.

It has been mentioned that dry films absorb water molecules when RH increases. The result of this phenomenon is the film swelling and the decrease of its refractive index (n_2_). Swelling causes the grating modulation (m) to increase. Considering the parameters m and n_2_, the first order intensity behavior can be calculated by using the formula reported above. Results can be seen in Figure 12. The modulation (m) deep was varied from 0 to 3 µm and the parameter is the refractive index (n_2_) that changed from 1.3 to 1.5. It is seen that when m = 0 the first order intensity is null because there is not a surface modulation. When modulation increases, first order intensity increases for all the plots, and presents a maximum intensity of 33%. Then it decreases and reaches a minimum value (zero). When the plot for n_2_ = 1.3 is chosen its full width at half maximum (FWHM) is about Δm = 1.28. By taking two points in the linear part of the plot we can find the slope to be −0.3686. Following the same method, we can find the slope of the linear part of the plot when n_2_ = 1.5 to be −0.59, and its FWHM Δm = 0.769. The slope of the plots shows us the change of intensity as a function of the change in modulation that is caused by changes in RH. This slope represents the sensitivity of the method. It is found that if refractive indices are closer to n_2_ = 1.5 the sensitivity is greater than when refractive indices are close to 1.3 as seen in Figure 12.

## 5. Diffraction Grating Fabrication Method and Investigation of Grating Profiles

**Figure 13:**
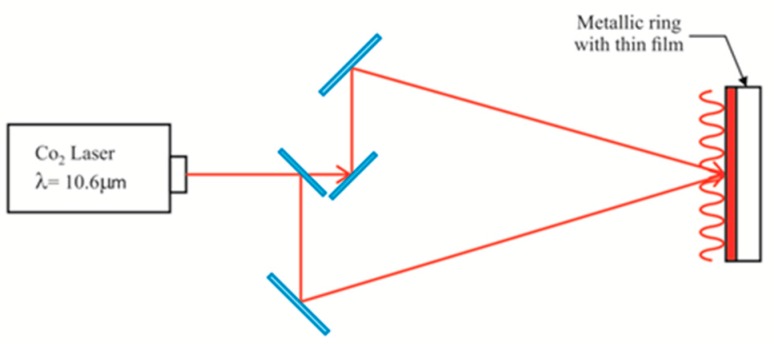
Infrared interferometric optical configuration to record the gratings on the surface of thin films. The sinusoidal red line represents the IR intensity field.

In previous work we used visible light to make the gratings [7]. Now a thermal method has been used to make the relief gratings [26]. Materials like gelatin and interpenetrated PVA/PAA films have been used. Thicknesses ranging from about 15 to 60 µm have been considered. To make the gratings a CO_2_ laser (λ = 10.6 µm) has been used. A two beam interference configuration was set up, Figure 13. This resulted in an IR sinusoidal interference pattern. When the IR light was absorbed by the films the spots in the sinusoidal pattern, corresponding to high intensities/constructive interference, partially melted the film so that some material was redistributed into the adjacent spots of lower intensity. The result was a sinusoidal relief.

**Figure 14:**
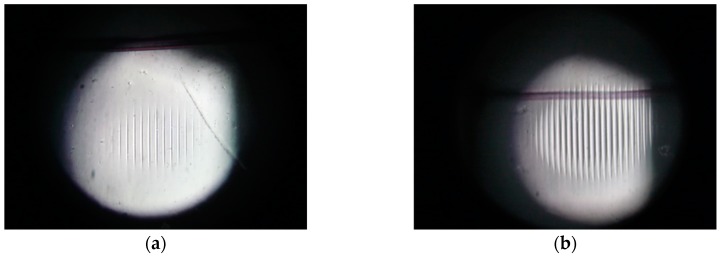
Photographs showing two fabricated gratings with exposure times of (**a**) 90 ms and (**b**) 140 ms. The distance between fringes is 263 µm.

The gratings fabrication method considered parameters like thin films thickness, beam intensity (10 W), and exposure times. Photographs in Figure 14 show two gratings made with gelatin with two exposure times, 90 ms and 140 ms. It is possible to notice the fringes widening as the exposure time increases.

To investigate the gratings relief two instruments were used, a profilometer and an interference microscope. The first is a mechanical device and the second relies on light interference.

**Figure 15:**
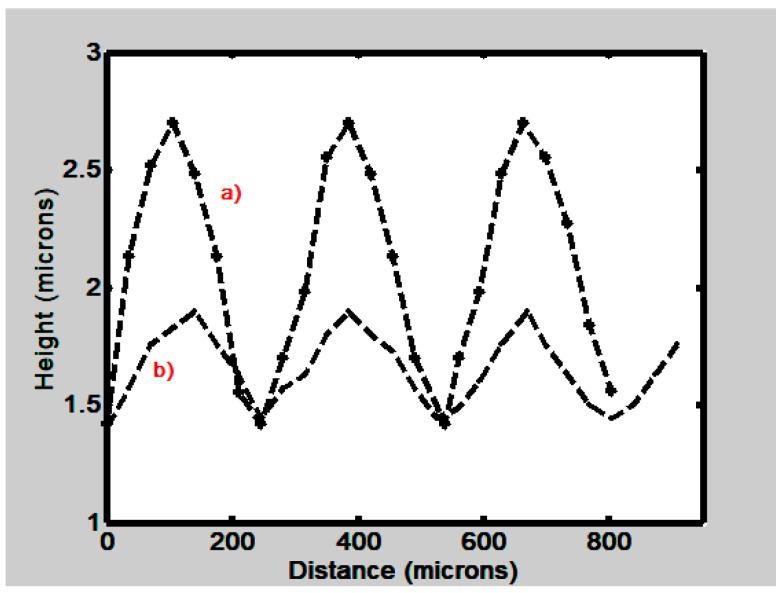
Profile height (µm) of a gelatin grating as a function of distance (µm). a) shows the profile after the gelatin thin film has absorbed water molecules. b) profile of a dry film.

It has been mentioned that the consequence of the absorption of water molecules by the films, with any of the materials mentioned in Section 2, is their swelling. To find the effect of swelling on the grating profile we performed the following experiment. At first the grating was scanned with the tip of the profilometer. At this time the film was dry. Then the film was let absorb water molecules from the atmosphere. A second scanning was done. The result can be seen in Figure 15. Two plots are displayed. One shows the profile (b) when the film was dry and the other (a) when the film swelled. This last one shows a deeper relief.

**Figure 16:**
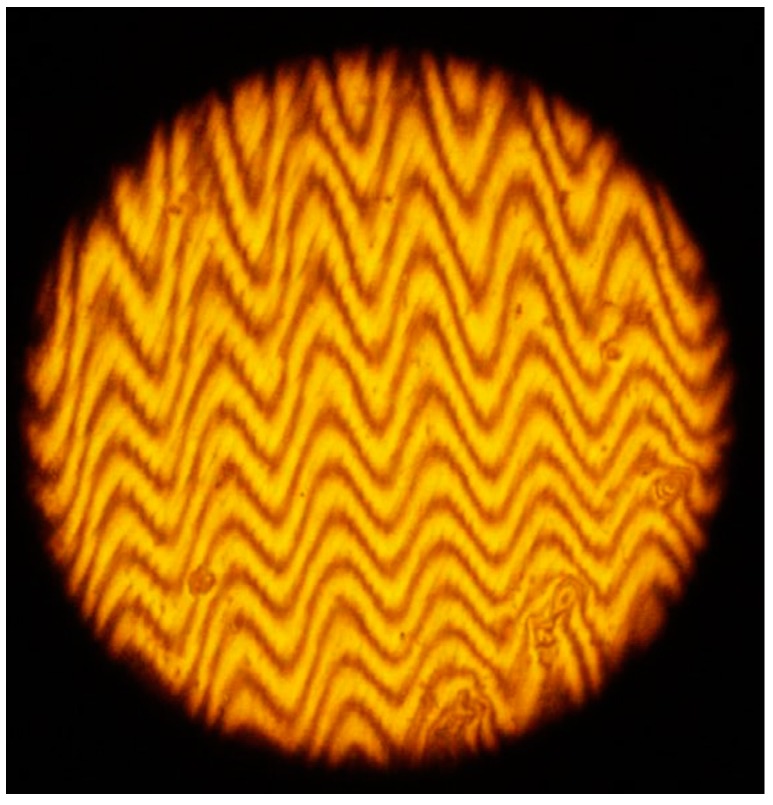
Gratings relief was investigated with an interference microscope. Photograph shows the relief of a PVA/PAA grating.

Besides the investigation of the grating surface with the profilometer an interference microscope was used. This microscope is based on a Michelson Interferometer. In one light trajectory a plane mirror is placed. In the other trajectory the object to be investigated is inserted, the grating. Then at the output of the interferometer the two beams interfere. The interference pattern shows the relief of the object. In Figure 16 an image is seen when a grating made with PVA/PAA film was investigated. The distance between crests was 630 µm. Each interference line shows a sinusoidal profile. 

## 6. Humidity Detection with Diffraction Gratings

It has been seen in Section 4 the theoretical description of relief diffraction gratings and in Section 5 their fabrication methods. Here we describe the use of diffraction gratings to detect RH. 

**Figure 17:**
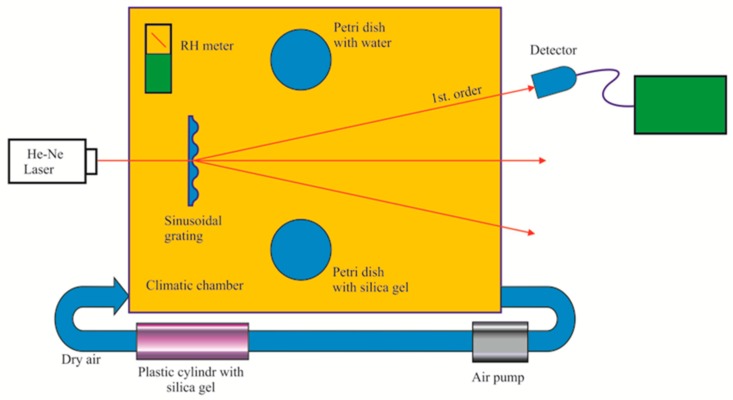
Schematic of a climatic chamber. Electronic RH instrument was used for calibration purposes. One Petri dish contained water and the other silica gel. Both dishes had lids that can be moved from outside.

To test the films as RH sensors a means to control the RH is needed. This instrument is named a climatic chamber. Figure 17 shows a schematic of the simple chamber made in our laboratory. A transparent plastic box with a lid was connected to an air pump that removed the humid air from the box. Then a hollow plastic cylinder filled with silica gel absorbed the water molecules and the dry air leaving the cylinder was pumped back into the box. Two petri dishes were placed inside the box. One contained water and the other silica gel. Both dishes had lids that can be displaced from outside the box. The former dish was used to increase the relative humidity and the latter helped to decrease the humidity together with the air pump. An electronic RH sensor [27] inside the box measured the humidity in the chamber as a means of calibration. Some mechanical mounts inside the box allowed holding the metallic rings with the thin films glued to them.

**Figure 18:**
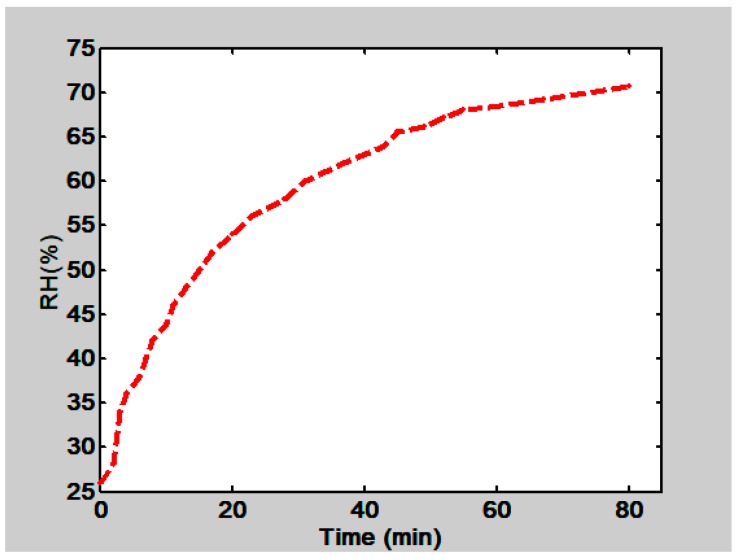
Behavior of RH in the climatic chamber as a function of time. RH was measured inside the climatic chamber when the petri dish lid that contained water was displaced.

An experiment was done to find the behavior of the RH when the petri dish lid, containing water, was displaced, Figure 18. It is seen that RH increases with time. At the end it tends to stabilize.

**Figure 19:**
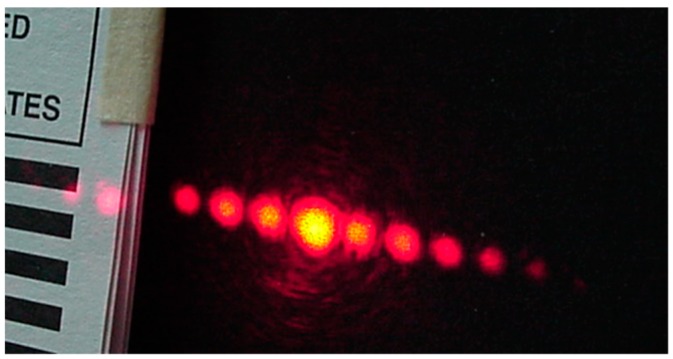
Diffracted orders given by a PVA/PAA grating.

**Figure 20:**
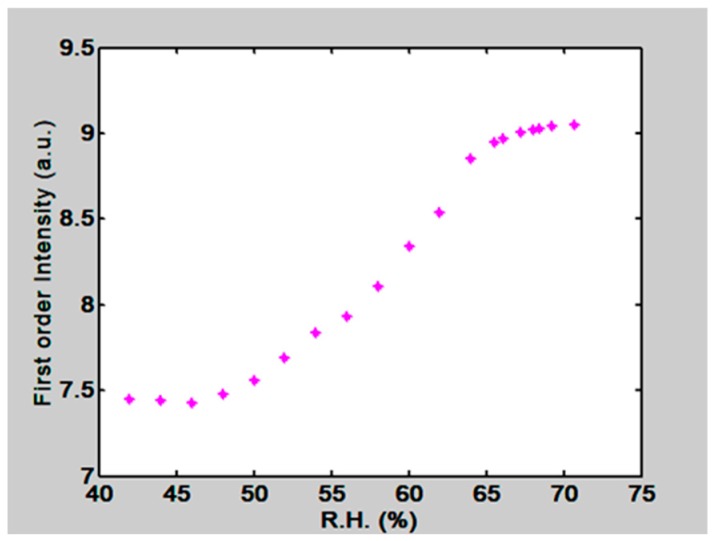
First order intensity as a function of RH in the climatic chamber. A PVA/PAA film with 50 µm thickness was used.

The gratings were placed in the climatic chamber and a beam of light coming from an intensity stabilized He-Ne laser (λ = 632.8 nm) illuminated the grating. At the far field, outside the box, some light spots (diffracted orders) were seen, Figure 19. Dry air was pumped into the climate chamber until a desired low RH was reached. Then the lid of the petri dish, that con tained water, was opened. Humidity began to increase and the first order intensity changed due to the materials swelling. The plot in Figure 20 shows the behavior of the first order intensity as a function of the RH for a PVA/PAA film. It is possible to see that the response is not immediate. RH needs to increase to let the film swell. The slope of this plot is 0.08/RH. The RH range is from about 45% to 65%.

**Figure 21:**
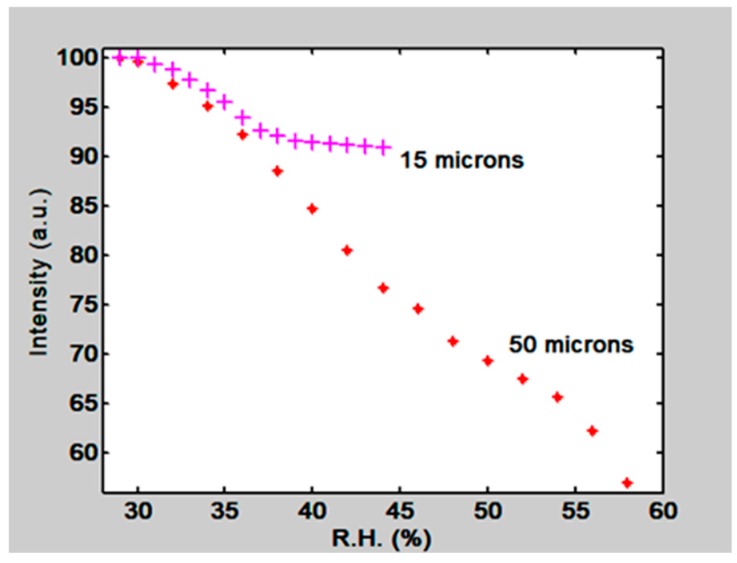
Normalized First order intensity, as a function of RH, for two gratings recorded in 15 µm and 50 µm thickness gelatin layers.

Besides the PVA/PAA film two gelatin films were tested. One had 15 µm thickness and the other 50 µm. Their normalized orders intensity behavior can be seen in Figure 21. For the 15 µm thickness film we have a slope of 0.91/RH, ranging from about 30% to 36% RH, and for the 50 µm thickness film a slope of 0.058/RH ranging from about 33% to 58% RH. Thus thin films are more sensitive than thick films but are useful in a shorter range. Thick films are less sensitive but useful in a wider range.

**Figure 22:**
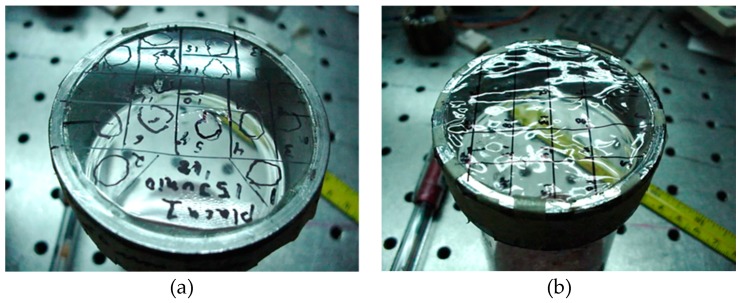
(**a**) shows the tense film surface when low humidity was present; (**b**) shows the film surface when humidity higher than about 70% was present. A loose surface can be seen.

We should mention that when RH reached a value of about more than 70% the films became loose, Figure 22. At this stage the orders light intensity values should not be considered because it is possible that light illuminates another part of the grating that could have another profile. 

## 7. Gels Hysteresis

**Figure 23:**
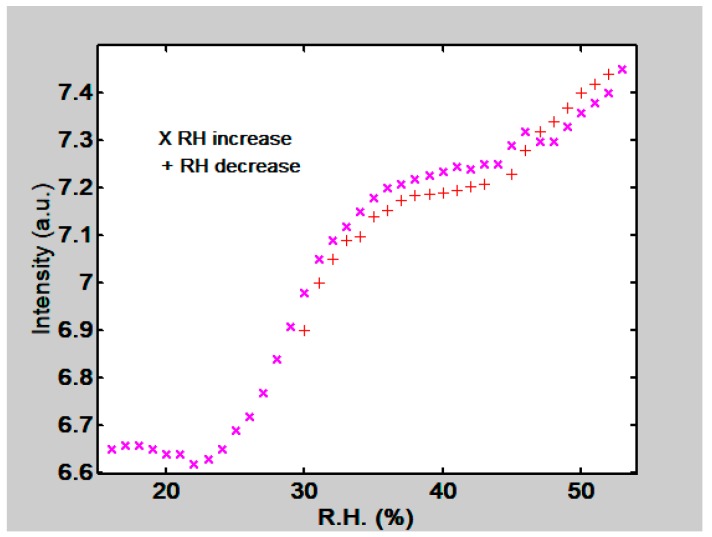
Behavior of first order intensity as a function of RH when it increases and decreases. Thin film thickness 15 µm.

The response of gelatin thin films when RH increased and decreased was investigated. A gelatin thin film with 15 µm thickness was used. First RH was increased by opening the petri dish that contained water in the climatic chamber. Then at a given RH it was closed and the petri dish that contained silica gel was opened. The behavior of the first order intensity can be seen in Figure 23. 

## 8. Conclusions

It has been presented the feasibility of using stimuli-responsive materials, like gelatin and PVA/PAA, in optical hygrometers. A study has been presented before [28] where they used gelatin as RH sensitive material. However, they considered the use of electronic devices to measure the capacitance of the gelatin films and not the optical and mechanical characteristics of films, refractive index and swelling, like we have done. Besides, the fabrication of those sensors [28] requires high tech instruments and methods like photolithography, spin coating, thermal physical evaporation and wet etching techniques. Characterization studies presented now comprise the behavior of gelatin and PVA/PAA films permeability and weight as a function of RH. Additionally diffraction gratings have been generated on the film’s surface. These gratings were placed in an optical configuration where light illuminated them and diffracted orders appeared. When RH surrounding the gratings changed the diffracted orders intensity also did it. Thus we have a calibration plot relating the light intensity as a function of RH.

These results should be taken as preliminary ones. Additional studies should be done by interdisciplinary research teams of chemist, physicists and materials science scientists to improve the response of films when used in hygrometers. In depth gelatin studies [29,30] should be considered.

A method to improve the sensitivity of the films to RH could be done by incorporating macropores in the films to let water molecules to be absorbed quickly. Also the use of PVA/gelatin interpenetrated films can be considered [31].

Regarding the Limit of Detection (LOD) of the proposed method we have the following comment. In Section 6 it was exposed that the slopes for plots in Figure 20 and Figure 21 (Intensity vs RH) were 0.08/RH and 0.091 /RH, If we suppose that the light detector can measure a minimum intensity with a value of 0.1 µW then the LOD, considering the slopes, will be 0.125 RHU. (Relative Humidity Unit) and 0.109 RHU respectively. However, it should be remembered that the method presented here can be modified according to the experimental conditions. We have variables that can be adapted to the needs. For example the RH sensitive materials, gelatin and interpenetrated polymers, can be modified. Besides the grating parameters can be changed. We have seen in Section 4 that it is possible to select the grating refractive index and this in turn will change the sensitivity of the method. Besides this it is possible to choose more powerful lasers and light detectors with greater sensitivities. Thus, given the RH measurement it is possible to select the material and the optical parameters that will better suit the experiment.

In the market there are many types of hygrometers. Each is useful for certain applications. For example we have mentioned one made with paper [7]. Also, there are others made with graphene oxide [8,32]. The first one relies on capacitance measurements and has a sensitivity of 2 pF/RH%. The one in reference [32] is based on frequency measurements and has a sensitivity of 719 Hz/RH%. The paper sensor is relatively easy to make. The GO sensor needs expensive, dedicated instruments, and expensive materials, and the fabrication method is difficult. The comparison of the RH sensor that we suggest with other sensors is difficult to make because it depends mainly on the application. 

By means of the optical method presented here it should be possible to study the response of materials to RH.

## Figures and Tables

**Table 1:** Band assignment of IR vibrational modes for gelatin, PVA, acrylic acid (AA), and PVA/PAA films.

Entry	N–H st.	–O–H st.	H–O–H st.	C–H st.	=C–H st.	C=O st.	C=C st.	CH_2_ b.	C–O b.	C–O st.	C–C st.
Gelatin	3793	3250	3595	2987	3125	1639	1535	1499	1480	1247	1090
PVA	-	3347	3443	3000		-	-	-	1554	1244	1085 1035 975
AA	-	3090	-	3000	2968 2902	1700	1434	-	1413	1298	1047 976
PVA/PAA 35 µm	-	3324	3443	3005	3094	1657–1618	1550	1340	1454	1244	1085 1035 976
PVA/PAA 30 µm	-	3324	3443	3005	3094	1657–1618	1550	1340	1454	1244	1085 1035 976

st. stretching; b. bending.
